# Vitamin D and Chronic Disorders: A Review of Metabolic and Cardiovascular Diseases

**DOI:** 10.3390/ph18101467

**Published:** 2025-09-29

**Authors:** Ewelina Młynarska, Wiktoria Lisińska, Katarzyna Hossa, Natalia Krupińska, Paulina Jakubowska, Jacek Rysz, Beata Franczyk

**Affiliations:** 1 Department of Nephrocardiology, Medical University of Lodz, 90-419 Lodz, Poland; 2 Department of Nephrology, Hypertension and Internal Medicine, Medical University of Lodz, 90-549 Lodz, Poland

**Keywords:** vitamin D receptor, diabetes mellitus, hypertension, chronic kidney disease, polycystic ovary syndrome, inflammation, calciferol, calcitriol

## Abstract

Vitamin D, long recognized for its essential role in calcium–phosphate balance and bone health, has increasingly been identified as a pleiotropic regulator of metabolic, cardiovascular, and renal function. Deficiency of vitamin D is widespread worldwide and has been linked to a higher risk of insulin resistance, type 2 diabetes, atherosclerosis, hypertension, and chronic kidney disease. Meta-analyses suggest that each 10 nmol/L (≈4 ng/mL) increase in serum 25-hydroxyvitamin D [25(OH)D] is associated with about a 4% lower risk of type 2 diabetes, whereas other analyses indicate an approximately 10% reduction in cardiovascular event risk per 10 ng/mL (≈25 nmol/L) increment in circulating 25(OH)D concentration. Clinical and epidemiological studies suggest that optimal 25(OH)D concentrations may protect against cardiometabolic and renal complications, though supplementation trials show heterogeneous outcomes depending on baseline vitamin D status, genetic background, and dosage. By synthesizing current knowledge, this work highlights vitamin D status as a potentially modifiable determinant of global disease burden and a target for preventive and therapeutic strategies.

## 1. Introduction

Due to their significant impact on mortality and the increasing burden on healthcare systems, metabolic diseases, cardiovascular diseases (CVDs), and chronic kidney disease (CKD) are among the major public health challenges of the 21st century. In 2022, cardiovascular diseases caused nearly 20 million deaths, accounting for approximately one-third of all global deaths. Over the past thirty years, the number of people living with diabetes has risen from 200 million to 830 million, illustrating the rapid global expansion of this disease [1]. Diabetes is a major risk factor for complications such as kidney failure, heart attack, and stroke; in 2021, diabetes and its renal consequences were responsible for over 2 million deaths worldwide [2]. CKD has also become an increasingly serious health issue—between 2000 and 2021, the number of deaths nearly doubled (a 95% increase), and CKD rose from the 19th to the 9th leading cause of global mortality. In the face of such serious threats, researchers are increasingly focusing on factors that may modify the risk of developing these diseases [3].

Vitamin D, long recognized for its role in calcium–phosphate homeostasis and bone metabolism, has increasingly been identified as a regulator of metabolic and cardiovascular health. Its influence extends to glucose regulation, immune function, vascular homeostasis, and inflammatory processes, linking deficiency to a wide range of lifestyle-relate [4]. This fat-soluble prohormone is essential for numerous physiological processes, with its primary function established in bone metabolism and calcium absorption. In recent years, several studies have highlighted how vitamin D, in addition to playing a fundamental role in calcium-phosphate homeostasis and consequently, in the proper functioning of the skeletal system in maintaining skeletal health, is implicated in a large number of extra skeletal physiological functions [5]. Beyond skeletal health, vitamin D exerts wide-ranging effects through endocrine, autocrine, and paracrine pathways that are vital for maintaining overall health [6].

Increasing evidence links vitamin D deficiency to various pathological conditions, underscoring its systemic actions. These include regulation of cell growth, differentiation, activation, and apoptosis, as well as roles in detoxification, antioxidant defense, neuroprotection, antimicrobial activity, immune regulation, and anti-inflammatory and anti-cancer mechanisms across different tissues [7]. Indeed, some lines of research have suggested a possible association between vitamin D homeostasis and glycemia, the immune system, inflammatory processes, and indirectly, the cardiovascular, neurological, gastrointestinal, reproductive systems, and oncological, psychiatric, infectious, metabolic, and immunological diseases [8]. Consequently, low serum vitamin D levels have been associated with chronic inflammatory conditions such as CVD, diabetes, insulin resistance (IR), metabolic syndrome, autoimmune and allergic disorders, infections, and malignancies. In particular, vitamin D appears to be a key factor in diabetes pathogenesis, where disruption of its signaling pathways increases the risk of autoimmune dysfunction and both microvascular and macrovascular complications [9].

**Figure 1:**
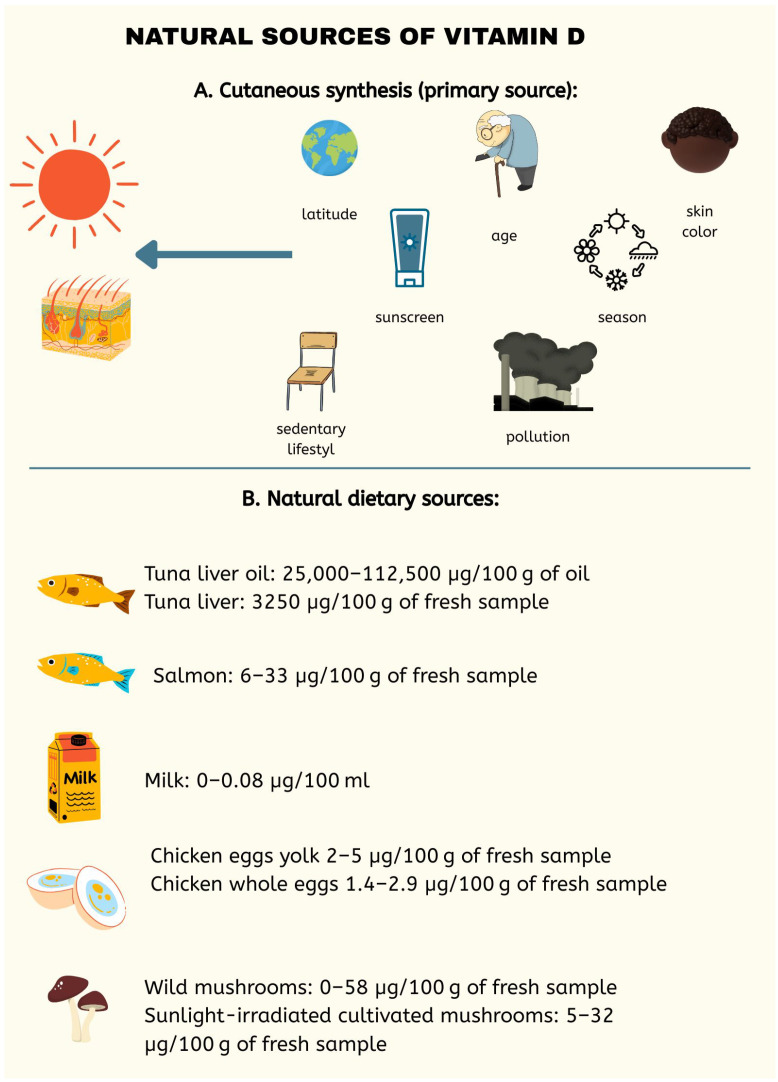
Main natural sources of vitamin D, emphasizing cutaneous synthesis through sunlight exposure as the primary source. Dietary sources, including fatty fish, eggs, milk, and certain mushrooms, are also presented, with their vitamin D content specified per 100 g of fresh sample or per 100 mL in the case of milk. Factors affecting vitamin D production were also considered including age, skin color, season, latitude, use of sunscreen creams [12].

Vitamin D is divided into classes numbered 2–6, with the two primary forms being D_2_ and D_3_, which differ by a methyl group at C24 and a double bond between C22 and C23 in D_2_. The synthesis of this compound occurs predominantly in the epidermis from the substrate 7-dehydrocholesterol (7-DHC) with the participation of UVB radiation (290–315 nm) and heat. In the skin, UVB breaks the B ring, forming pre-vitamin D_3_, which is then rearranged into vitamin D_3_ by heat [10,11]. In this way, the D_3_ form (cholecalciferol) is produced, which can also be obtained from animal-derived foods, such as fish liver oils, fatty fish (including salmon, sardines, herring, and mackerel), and egg yolk (Figure 1). Processed foods fortified with vitamin D_3_ include dairy products, as well as orange juice and breakfast cereals. In contrast, vitamin D_2_ (ergocalciferol) is found in plant sources and even in fungi [10,12]. A summary is shown in Figure 1.

Activation of vitamin D_3_ involves two hydroxylations. First, vitamin D is metabolized in the liver by CYP2R1, producing the main circulating form, 25-hydroxyvitamin D [25(OH)D], which is then activated in the kidneys by CYP27B1 to 1,25-dihydroxyvitamin D [1,25(OH)_2_D] [13]. This process is regulated by calcium and phosphorus levels, the concentration of 1,25(OH)_2_D, and parathyroid hormone (PTH). Transport in the blood occurs via chylomicrons and vitamin-D-binding protein (DBP) [11]. The active form of vitamin D, 1,25(OH)_2_D, exerts its biological effects primarily through the nuclear vitamin D receptor (VDR), which is widely distributed in the body and regulates the expression of more than a thousand genes. VDR forms a heterodimer with retinoid X receptors (RXR), binds to vitamin-D-responsive elements (VDREs) sequences in gene promoters, and, upon ligand binding, recruits coactivators, leading to transcriptional activation. This mechanism plays a role in the pathogenesis of many diseases, including obesity.

Vitamin D can also rapidly activate signaling pathways such as phospholipases C and A_2_, phosphatidylinositol 3-kinase (PI3K), protein kinase A (PKA), and mitogen-activated protein kinases (MAPK), as well as induce the opening of Ca^2+^ and Cl^−^ channels via the membrane receptor protein disulfide-isomerase A3 (PDIA3).

At the epigenetic level, vitamin D modulates DNA methylation, histone modifications, and the regulation of microRNA expression, including the reduction in pro-inflammatory microRNA levels in adipocytes [14]. Vitamin D molecules, through their nuclear receptor VDR, influence gene expression through epigenetic modifications, including transcription factor binding, histone modifications, regulation of chromatin accessibility, and reorganization of 3D chromatin architecture across a wide range of tissues and cell types [15]. The immunological effects of vitamin D are mediated through its interaction also with VDR, present on macrophages, dendritic cells, and T and B lymphocytes. Vitamin D acts as a true “molecular regulator”, influencing a wide range of essential biological functions [16]. Vitamin D enhances innate immunity by improving the function of phagocytic and dendritic cells, while also modulating adaptive immunity. It supports regulatory T cells and Th2 cells, and inhibits excessive activation of Th1, Th17, and B cells [17]. Given the association between vitamin D and immune function, it has been hypothesized that vitamin D deficiency predisposes individuals to autoimmune diseases such as rheumatoid arthritis (RA), systemic lupus erythematosus (SLE), antiphospholipid syndrome (APS), Hashimoto’s thyroiditis (HT), and multiple sclerosis (MS) [18].

The purpose of this review is therefore to evaluate the role of vitamin D in the prevention and progression of cardiometabolic diseases and chronic kidney disease, with particular emphasis on molecular mechanisms, metabolic pathways, and available clinical evidence on supplementation [19]. By summarizing current knowledge, this work aims to provide a comprehensive understanding of how vitamin D status may serve as a modifiable factor in reducing disease burden and improving patient outcomes.

## 2. Metabolic Disorders

### 2.1. Vitamin D in Type 1 Diabetes Mellitus

Type 1 diabetes mellitus (T1DM) is an autoimmune disease characterized by the immune-mediated destruction of pancreatic β-cells, resulting in absolute insulin deficiency [20]. Vitamin D plays an important immunomodulatory role in T1DM pathogenesis and prevention, particularly during infancy and early childhood, when it supports immune maturation and tolerance [21,22]. Through VDR, expressed in various immune cells, 1,25(OH)_2_D regulates both innate and adaptive immunity by downregulating antigen presentation (MHC II, CD40, CD80, CD86) in monocytes/macrophages, inducing tolerogenic dendritic cells, enhancing regulatory T cell activity, and suppressing pro-inflammatory Th1/Th17 responses and β-cell-directed chemokines (e.g., CCL2, CXCL10) [23]. It also inhibits B cell proliferation and antibody production and promotes anti-inflammatory macrophage polarization (M1→M2) via the VDR to peroxisome proliferator-activated receptor gamma (PPARγ) pathway [24]. In animal models, high-dose calcitriol or vitamin D analogs reduce islet inflammation and preserve β-cell function. Epidemiological studies link higher early-life vitamin D intake and serum 25(OH)D levels to reduced islet autoimmunity and T1DM risk, while deficiency is prevalent in affected individuals. However, clinical trial results are mixed: some report improved C-peptide levels and reduced insulin needs, whereas others show no significant benefit after onset, likely due to irreversible β-cell loss at diagnosis. Genetic studies on VDR polymorphisms suggest only a modest effect on disease susceptibility. Overall, adequate vitamin D status may lower the risk of T1DM development, but its therapeutic value post-diagnosis remains uncertain, warranting further well-powered randomized trials [20].

Meta-analyses conducted by Low et al. suggest that vitamin D supplementation does not significantly reduce the risk of developing islet autoimmunity (IA) or T1DM, nor does it affect progression from IA to T1DM [25]. While some individual studies, such as the EURODIAB study, reported a potential protective effect, the results across the literature are inconsistent due to heterogeneity in study populations, supplementation timing, dosage, and outcome definitions [26]. These findings indicate that, although vitamin D may play a role in immune regulation, its impact on T1DM risk is limited and likely influenced by genetic, environmental, and individual factors [27]. However, investigating vitamin D supplementation in type 1 diabetes mellitus (T1DM) suggests a possible protective role, particularly in preserving β-cell function, as indicated by improved fasting C-peptide levels. These findings must be interpreted with caution, as the evidence is limited by small sample sizes, heterogeneity in vitamin D formulations, and overall low to very-low quality ratings. Most included studies showed concerns or high risk of bias, and inconsistencies across outcomes further weaken the reliability of the results. While preliminary data hint at a beneficial role of vitamin D in slowing T1DM progression, current evidence from meta-analyses is insufficient to support clinical recommendations, highlighting the need for larger, well-designed trials [28].

### 2.2. Vitamin D in Type 2 Diabetes Mellitus

Type 2 diabetes mellitus (T2DM) is a multifactorial metabolic disease characterized by chronic hyperglycemia resulting from a combination of peripheral insulin resistance, progressive pancreatic β-cell dysfunction, and impaired incretin action [29,30]. It is closely associated with obesity, chronic low-grade inflammation, oxidative stress, and alterations in lipid metabolism [31]. Chronic inflammation plays a central role in the development and progression of insulin resistance (IR), which is a hallmark feature of multiple metabolic disorders such as T2DM, metabolic syndrome, non-alcoholic fatty liver disease (NAFLD), and polycystic ovary syndrome (PCOS), and it is intricately linked with obesity, dyslipidemia, and chronic low-grade systemic inflammation [32]. Adipose tissue, particularly visceral fat, acts as an endocrine organ, releasing pro-inflammatory cytokines such as tumor necrosis factor-alpha (TNF-α), interleukin-6 (IL-6), and interleukin-1 beta (IL-1β), as well as chemokines that recruit macrophages and other immune cells. These inflammatory mediators interfere with insulin receptor signaling by activating serine kinases, promoting serine phosphorylation of insulin receptor substrates, impairing glucose transport, and disrupting insulin-mediated intracellular signaling [33]. Moreover, chronic inflammation contributes to pancreatic β-cell dysfunction, promoting apoptosis and impairing insulin secretion while also exacerbating oxidative stress and promoting the accumulation of advanced glycation end-products (AGEs), further aggravating IR and T2DM [34].

**Figure 2:**
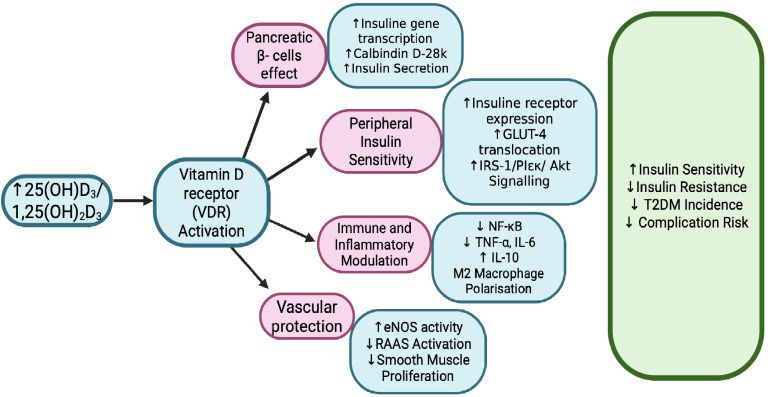
Vitamin D Signaling and Type 2 Diabetes Risk [33,34,39,44]. Vitamin D influences multiple pathways involved in type 2 diabetes, including insulin secretion, insulin sensitivity, inflammation, and vascular function. It enhances pancreatic β-cell function, promotes glucose uptake in muscle and fat, and shifts immune responses toward an anti-inflammatory state. Additionally, it supports vascular health by modulating RAAS and improving endothelial function. 25(OH)D_3_—25-hydroxyvitamin D_3_ (calcidiol); 1,25(OH)_2_D_3_—1,25-dihydroxyvitamin D_3_ (calcitriol); VDR—Vitamin D receptor; GLUT-4—Glucose transporter type 4; IRS-1—Insulin receptor substrate 1; PI3K—Phosphoinositide 3-kinase; Akt—Protein kinase B; NF-κB—Nuclear factor kappa-light-chain-enhancer of activated B cells (a transcription factor regulating inflammation); TNF-α—Tumor necrosis factor alpha; IL-6—Interleukin 6; IL-10—Interleukin 10 (anti-inflammatory cytokine); eNOS—Endothelial nitric oxide synthase; RAAS—Renin–angiotensin–aldosterone system; T2DM—Type 2 diabetes mellitus.

While traditionally recognized for its role in calcium and phosphate homeostasis, vitamin D has emerged as a potential modulator of glucose homeostasis, insulin action, and inflammatory responses [35]. The active form, 1,25(OH)_2_D, exerts its biological effects through binding to VDR, a nuclear transcription factor expressed in numerous tissues integral to glucose metabolism, including pancreatic islets, skeletal muscle, adipose tissue, and the liver [36]. This broad tissue distribution suggests that vitamin D may influence multiple pathogenic pathways in T2DM: ranging from β-cell preservation and insulin signaling enhancement to anti-inflammatory and vasculoprotective effects [13]. Since VDR mediates vitamin D’s effects, we looked at the Fok1 (rs2228570) variant, which produces a slightly shorter receptor protein. Nevertheless, analyses including gene expression studies and a meta-analysis found no clear association between this variant and T2DM [37]. At the pancreatic level, vitamin D facilitates insulin biosynthesis and secretion by modulating intracellular calcium flux, which is essential for insulin granule exocytosis [38]. It regulates the expression of genes encoding insulin and insulinotropic enzymes, such as calbindin, thereby enhancing β-cell responsiveness to glucose. In insulin-sensitive tissues, VDR activation increases the transcription of insulin receptor genes and insulin receptor substrate proteins (IRS-1 and IRS-2), promoting effective downstream signaling via the PI3K and Akt pathways [39]. Vitamin D also upregulates Glucose Transporter Type 4 (GLUT4) expression in skeletal muscle and adipocytes, enhancing peripheral glucose uptake [40]. Moreover, vitamin D exerts potent anti-inflammatory actions by inhibiting nuclear factor kappa B (NF-κB) activation, downregulating pro-inflammatory cytokines (TNF-α, IL-1β, IL-6), and suppressing chemokine production, which collectively reduce macrophage recruitment and adipose tissue inflammation [41]. It shifts the immune balance toward a more tolerogenic state by promoting the differentiation of regulatory T cells and reducing Th1/Th17 pro-inflammatory responses [29]. A study conducted by Arfian N. et al. in diabetic mice demonstrated that vitamin D (calcitriol) treatment reduced lung inflammation. Diabetic mice showed increased inflammatory markers, including NF-κB, IL-6, monocyte chemoattractant protein-1 (MCP-1), and macrophage infiltration (CD68), but these were significantly lower in vitamin-D-treated groups. These findings suggest that vitamin D can attenuate inflammatory responses, likely through its immunomodulatory and anti-oxidative effects, offering protective benefits against diabetes-related lung injury [42]. Moreover, vitamin D plays a role in regulating immune system activity, particularly by enhancing antimicrobial defenses such as cathelicidin production in monocytes and Paneth cells, which helps protect against infections and reduce intestinal inflammation. Its signaling through VDR and activation of Toll-like receptors promotes macrophage antimicrobial effects, while mTOR-mediated pathways aid in reducing airway inflammation via increased autophagy. Although some studies suggest vitamin D supplementation may lower the risk of respiratory infections, results remain inconsistent, highlighting the need for larger, well-designed trials [43]. In adipose tissue, vitamin D inhibits preadipocyte differentiation, modulates adipokine secretion toward a more insulin-sensitizing profile (increasing adiponectin, reducing resistin), and attenuates oxidative stress through upregulation of antioxidant enzymes [44,45]. The schematic in Figure 2 illustrates the main mechanisms by which vitamin D influences the risk of developing type 2 diabetes.

Vitamin D deficiency, commonly defined as serum 25(OH)D concentrations below 50 nmol/L, is highly prevalent among individuals with T2DM, with reports indicating prevalence rates between 60 and 90%, depending on the population studied [46]. Observational studies have consistently demonstrated inverse correlations between serum 25(OH)D levels and markers of insulin resistance, such as homeostatic model assessment–insulin resistance (HOMA-IR), as well as positive correlations with β-cell function indices homeostasis model assessment of b function (HOMA-B) [47].

Meta-analyses of longitudinal studies indicate that each 10 nmol/L increase in serum 25(OH)D is associated with an approximate 4% reduction in T2DM incidence. Research by Wenwen Jiang et al. found that in patients with T2DM, serum 25(OH)D levels were positively correlated with time in range, with this relationship being markedly stronger than that observed between 25(OH)D and hemoglobin A1c (HbA1c) [48]. Furthermore, 25(OH)D concentrations showed an inverse association with glycemic variability metrics and a positive association with measures of glucose-stimulated insulin secretion. These observations indicate that vitamin D insufficiency may adversely affect glucose regulation and β-cell secretory function in T2DM. This study highlights the potential of vitamin D supplementation as a supportive approach in T2DM management, given its favorable associations with glycemic stability and β-cell function [49]. Although interventional studies have yielded mixed results, several randomized controlled trials (RCTs) in vitamin-D-deficient or prediabetic populations have demonstrated improvements in fasting plasma glucose, HbA1c, insulin sensitivity, and β-cell function—particularly with higher doses (≥2000 IU/day) administered over at least six months [50]. These findings reinforce the rationale for considering vitamin D supplementation in clinical practice and underscore the importance of including time in range, alongside HbA1c, as a key endpoint in future trials assessing its impact on glycemic control [48].

A meta-analysis conducted by Zhang XJ et al. of 40 RCTs involving over 4000 adults with T2DM showed that vitamin D supplementation, particularly at higher doses (>4000 IU/day), significantly increased serum 25(OH)D concentrations and reduced fasting blood glucose, while lower daily doses were more effective in improving HbA1c, suggesting a dose- and duration-dependent effect [51]. However, evaluation and effects of vitamin D supplementation on glycemic control and inflammation in T2DM showed modest short-term benefits, including reductions in insulin levels, HbA1c, high-sensitivity C-Reactive Protein (hs-CRP), and HOMA-B at 12 weeks, though many of these effects diminished by 24 weeks. While vitamin D supplementation improves serum vitamin D levels and may offer short-term metabolic and anti-inflammatory benefits, longer and better-designed trials are needed to clarify its sustained role in T2DM management [52]. This meta-analysis, which enrolled 39 RCTs, demonstrated that vitamin D supplementation significantly improved glycaemic markers in T2DM patients, with notable reductions in fasting blood glucose (FBG) (−0.46 mmol/L), HbA1c (−0.30%), HOMA-IR (−0.44), and fasting insulin (−1.06 µU/mL). The benefits were more pronounced in patients with vitamin D deficiency, HbA1c ≥ 8%, or overweight/obese status. However, limitations such as high heterogeneity across studies, varying supplementation doses (average 3993–4700 IU/d), short follow-up in many trials, and unmeasured confounders like sun exposure and concomitant medications reduce certainty. Despite these concerns, the findings highlight vitamin D supplementation as a promising adjunct for glycaemic control in T2DM, warranting further long-term, standardized trials [53].

Vitamin D deficiency in T2DM patients is linked to increased prevalence and severity not only of microvascular complications such as diabetic retinopathy, nephropathy, and neuropathy but also of macrovascular outcomes, including myocardial infarction, stroke, and peripheral artery disease [54,55]. These associations may reflect vitamin D’s anti-inflammatory, anti-fibrotic, and endothelial-protective actions. A study conducted by Tan J. et al. showed that vitamin D may have a protective effect in T2DM complicated with lower extremity arterial disease (LEAD) [55]. Patients with T2DM and LEAD had lower serum 25(OH)D levels, and those receiving vitamin D supplementation had higher vitamin D levels and reduced rates of arterial stenosis and occlusion, especially in the inferior knee artery, suggesting that adequate vitamin D may help mitigate vascular complications in T2DM [56]. Moreover, a study analyzing 628 Chinese patients with T2DM found that lower serum 25(OH)D levels were associated with the presence and severity of lower extremity arterial plaques. Patients with more severe plaques had significantly lower vitamin D levels, and vitamin D deficiency was identified as an independent risk factor for plaque formation. These findings suggest that maintaining adequate vitamin D levels may help reduce the risk and progression of lower extremity vascular complications in T2DM [57]. Emerging evidence suggests that vitamin D may also play a role in sexual dysfunction associated with T2DM. A meta-analysis found that men with T2DM and erectile dysfunction had significantly lower serum vitamin D levels compared to diabetic men without erectile dysfunction, indicating that vitamin D deficiency may be a contributing risk factor [58]. In female models of diabetes, severe hyperglycemia—more common in T1DM—was associated with decreased sexual response, while T2DM produced milder effects. These findings highlight that vitamin D status and glycemic control could influence sexual health in both men and women with diabetes, suggesting potential avenues for prevention and therapeutic intervention [59]. Reduced serum 25(OH)D levels are significantly associated with an increased risk and severity of diabetic peripheral neuropathy (DPN) in T2DM. In this study, both 25(OH)D and 25(OH)D_3_ were lower in the DPN group, and deficiency was more common among affected patients. Higher 25(OH)D levels were linked to a lower incidence of DPN, and logistic regression identified 25(OH)D as an independent protective factor. ROC analysis suggested that specific 25(OH)D and 25(OH)D_3_ thresholds may help predict neuropathy risk, highlighting the potential role of adequate vitamin D status in preventing DPN [60].

### 2.3. Vitamin D in Gestational Diabetes Mellitus

Pregnancy induces profound physiological adaptations in maternal metabolism to support fetal growth, including marked changes in vitamin D metabolism. The rise in glomerular filtration rate (GFR) during pregnancy increases urinary calcium excretion. Moreover, total serum calcium and phosphate levels decline, ionic calcium remains stable due to enhanced intestinal calcium absorption. To maintain maternal–fetal calcium balance, pregnant women require approximately 1200 mg of calcium daily. While parathyroid hormone is the primary regulator of calcium and bone metabolism outside of pregnancy, vitamin D assumes a dominant role during gestation. Levels of 1,25(OH)_2_D increase two- to threefold, promoting greater intestinal calcium absorption to support fetal skeletal mineralization [61]. Serum concentrations of the active metabolite 1,25(OH)_2_D begin rising in early gestation and peak during the third trimester at levels two- to three-fold higher than in non-pregnant women, reflecting increased maternal and placental demands [62]. During pregnancy, vitamin D metabolism undergoes extraordinary changes that extend beyond its classic role in calcium absorption [63]. Normally, vitamin D from diet or sunlight is metabolized in the liver to 25(OH)D, which is then converted in the kidneys to its active form, calcitriol. However, pregnancy triggers a dramatic increase in circulating calcitriol levels, beginning soon after placental implantation. Vitamin D is now recognized as a pleiotropic hormone with biological effects extending across multiple organ systems. In addition to its established role in calcium regulation and skeletal integrity, where deficiency manifests as rickets in children and osteomalacia in adults, it modulates the expression of numerous genes governing immune function, cellular proliferation, differentiation, and metabolism. These broad regulatory functions provide the basis for accumulating evidence that links inadequate vitamin D status to an increased risk of diverse chronic diseases, including autoimmune, cardiovascular, infectious, metabolic, neurological, psychiatric disorders, and several malignancies [64]. While the maternal kidneys produce most of this excess, the placenta also plays an active role by taking up maternal 25(OH)D, converting it to calcitriol, and influencing gene expression important for placental function [65]. During pregnancy, vitamin D metabolism undergoes profound adaptations distinct from the non-pregnant state. The mother is the sole source of vitamin D for the fetus, and maternal deficiency directly results in fetal and neonatal deficiency [66]. Key changes include a marked rise in maternal serum 1,25(OH)_2_D, driven by increased renal and placental CYP27B1 activity, higher vitamin-D-binding protein levels, and reduced catabolism, while maternal 25(OH)D remains relatively constant. The placenta contributes significantly to vitamin D activation, as both maternal and fetal tissues express VDR and CYP27B1 [67]. Elevated 1,25(OH)_2_D enhances intestinal calcium absorption to meet fetal skeletal mineralization needs, creating a physiological dissociation between vitamin D and calcium homeostasis. Evidence suggests that optimal maternal vitamin D status during pregnancy requires circulating 25(OH)D concentrations of at least 40 ng/mL to sustain adequate 1,25(OH)_2_D production [68]. These processes may affect not only calcium homeostasis but also immune modulation, fetal development, and protection against complications such as pre-eclampsia [69]. Emerging evidence shows that vitamin D’s impact in pregnancy includes epigenetic changes in the placenta, suggesting that optimal maternal vitamin D status could have lasting effects on maternal and fetal health [63].

Gestational Diabetes Mellitus (GDM) is now a common condition, affecting approximately 15–25% of pregnancies globally, characterized as any degree of glucose intolerance that is first detected during pregnancy [70]. The pathogenesis of GDM is multifactorial, involving genetic predisposition (e.g., family history of diabetes, insulin resistance, immune dysregulation), environmental factors (dietary habits, β-cell damage), and metabolic stressors during pregnancy. Chronic low-grade inflammation and oxidative stress have been implicated as key drivers, with elevated inflammatory markers in early pregnancy correlating with higher GDM risk and hyperglycemia. In GDM, increased oxidative stress and diminished antioxidant defenses contribute to β-cell injury, apoptosis, and impaired insulin secretion. Vitamin D deficiency appears to exacerbate these processes through multiple mechanisms: activating the NF-κB pathway in pancreatic tissue, promoting pro-inflammatory cytokine production, increasing intracellular Ca^2+^ and reactive oxygen species (ROS) levels, and inducing β-cell death. Additionally, vitamin D modulates epigenetic regulation by preventing hypermethylation of genes protective against diabetes via upregulation of DNA demethylases. Its role in calcium homeostasis also intersects with obesity risk, another contributor to GDM pathophysiology. Table 1 presents variants of the VDR gene that have been increasingly recognized as potential contributors to the etiology of gestational diabetes mellitus. Polymorphisms in the VDR gene may alter receptor activity and downstream signaling, thereby influencing glucose metabolism, insulin secretion, and immune modulation during pregnancy. Studies have identified several single-nucleotide polymorphisms (SNPs), including: rs7975232 (ApaI), rs2228570 (FokI), rs1544410 (BsmI), and rs731236 (TaqI), that are associated with a higher risk of GDM in different populations [71]. In particular, rs2228570 has been linked to increased susceptibility, while rs1544410 may influence insulin secretion in affected women. Meta-analyses further support the associations of rs7975232 and rs2228570 with GDM risk. However, not all findings are consistent, as some variants, such as rs739837, show no association with disease development. These data suggest that VDR gene polymorphisms may contribute to the multifactorial pathogenesis of GDM, although their impact likely depends on genetic background, ethnicity, and environmental interactions [72,73].

Poor glycemic control in GDM increases the risk of long-term consequences to offspring: gestational hypertension, preeclampsia, macrosomia, neonatal hypoglycemia, congenital anomalies, glucose intolerance, obesity, endocrine and cardiovascular morbidity, neurodevelopmental and neuropsychiatric morbidity (autism spectrum disorder, epilepsy, and eating disorders), ophthalmic disease and predisposes women to T2DM, metabolic syndrome, ophthalmic morbidity (e.g., glaucoma, diabetic retinopathy, retinal detachment), renal diseases (e.g., hypertensive renal disease without renal failure, hypertensive renal disease with renal failure, chronic renal failure, and end-stage renal disease), CVD later in life [75,76].

However, clinical studies evaluating vitamin D supplementation in pregnancy have yielded inconsistent results. While some data suggest that replacement may lower the risk of GDM, other trials and meta-analyses report no significant benefit. These conflicting findings indicate that GDM has a multifactorial etiology and that vitamin D alone may not be sufficient to prevent its development. Further large-scale, dose–response studies are needed to clarify whether optimizing maternal vitamin D status can effectively reduce the incidence of GDM [77,78]. Epidemiological data consistently link low serum 25(OH)D levels in early pregnancy with increased GDM risk, and supplementation studies provide mechanistic and clinical support for this association. Vitamin D enhances insulin secretion, upregulates insulin receptor expression, and improves peripheral insulin sensitivity, thereby lowering fasting plasma glucose and HOMA-IR. It also exerts antioxidant effects that protect β-cells from oxidative damage [79]. RCTs and meta-analyses have reported that vitamin D supplementation in women with GDM significantly reduces fasting plasma glucose, insulin levels, and insulin resistance, and improves lipid profiles: raising high-density lipoprotein (HDL) cholesterol while lowering total cholesterol and low-density lipoprotein (LDL) cholesterol [80]. Furthermore, supplementation has been associated with reduced rates of cesarean section, postpartum hemorrhage, maternal hospitalization, and neonatal complications including macrosomia, hypoglycemia, hyperbilirubinemia, fetal distress, polyhydramnios, and preterm delivery, defined as gestational age < 37 week at delivery [81,82]. However, some meta-analyses report heterogeneity in lipid outcomes, possibly due to short intervention durations or differences in baseline vitamin D status. Lower maternal serum 25(OH)D levels in late pregnancy were linked to greater fetal fat accumulation. This suggests that maternal 25(OH)D could serve as an early indicator of fetal adiposity, particularly in pregnancies affected by diabetes [83]. Strategies aimed at raising maternal 25(OH)D concentrations in women with GDM through dietary changes or supplementation may help reduce fetal fat deposition, potentially preventing excessive newborn adiposity and lowering the risk of childhood obesity and early-onset metabolic syndrome later in life [84].

### 2.4. Vitamin D in Polycystic Ovary Syndrome

Polycystic ovary syndrome (PCOS) is a complex endocrine disorder marked by high androgens, chronic anovulation, and polycystic ovaries, with causes that are not fully understood [85]. IR is common, driving high insulin levels that lower sex hormone binding globulin (SHBG) production and stimulate ovarian androgen synthesis, creating a self-reinforcing cycle. Anti-Müllerian hormone (AMH), often elevated in PCOS, is linked to both androgen excess and insulin resistance. Central obesity, more frequent in PCOS, further worsens metabolic dysfunction [86].

Vitamin D regulates female reproductive function via steroidogenesis and hormones such as AMH, follicle-stimulating hormone (FSH), and progesterone in granulosa cells, as well as glucose homeostasis in pancreatic β-cells [87]. The VDR, which is a ligand-dependent transcription factor in the steroid/thyroid hormone receptor superfamily, mediates vitamin D’s actions. The VDR gene, located on chromosome 12q13.11, contains 14 exons encoding a 427-amino-acid protein [88]. Five common single-nucleotide polymorphisms (SNPs) have been identified: ApaI (rs7975232) and BsmI (rs1544410) in intron 8, Cdx2 (rs11568820) in exon 1, FokI (rs10735810) in exon 2, and TaqI (rs731236) in exon 9 [89]. Understanding VDR polymorphisms’ links to PCOS and infertility is crucial for improving diagnosis, treatment, and management. While evidence remains mixed, previous meta-analyses suggest associations between certain polymorphisms, particularly ApaI, BsmI, and FokI, and PCOS development. The present meta-analysis examined 5618 participants across five key VDR SNPs (ApaI, BsmI, Cdx2, FokI, TaqI) to assess PCOS risk [90].

Findings indicate significant associations between ApaI (rs7975232) and BsmI (rs1544410) polymorphisms and increased susceptibility to PCOS and infertility. The results for Cdx2 (rs11568820) were inconsistent, reflecting potential influences of ethnicity, BMI, environmental factors, and vitamin D status. FokI (rs22228570) showed no significant association, whereas TaqI (rs731236) was linked to increased risk. Subgroup analyses highlighted the modifying effects of body mass index (BMI) and ethnicity on the relationship between VDR polymorphisms and PCOS risk. These results reinforce the role of specific VDR polymorphisms in modulating PCOS susceptibility and underscore the need for further studies incorporating diverse populations and detailed data on confounders such as vitamin D levels, lifestyle, and environmental exposures [89].

Mechanistically, vitamin D may exert effects through multiple pathways: activating VDR in reproductive tissues to modulate steroidogenesis, regulating insulin receptor expression and pancreatic β-cell function, mitigating the harmful effects of advanced glycation end products (AGEs) on ovarian granulosa cells, and influencing genes involved in inflammation, lipid metabolism, and follicular development. Genetic variations in VDR also appear to influence PCOS susceptibility, metabolic profile, and insulin resistance, though findings are inconsistent [91].

Deficiency in vitamin D is common among women with PCOS, affecting approximately 67–85% of patients, and has been linked to key characteristics of the disorder, including insulin resistance, ovulatory dysfunction, hyperandrogenism, hirsutism, obesity, systemic inflammation, and increased cardiovascular risk [92]. Observational studies indicate that serum 25(OH)D levels correlate positively with SHBG and negatively with androgen levels, BMI, and inflammatory markers [93,94]. Clinically, vitamin-D-deficient women with PCOS are more likely to exhibit glucose intolerance, hypertension, dyslipidemia, elevated CRP, and lower HDL cholesterol. Moreover, deficiency has been associated with impaired folliculogenesis, reduced fertility, and lower live birth rates after ovarian stimulation, although results are not entirely consistent across studies [86]. Low vitamin D has also been linked to higher AMH levels in adolescents with PCOS, suggesting a potential role for combined AMH and 25(OH)D measurements in early risk assessment [6]. Additionally, vitamin D deficiency may compromise bone health, increasing long-term fracture risk due to the combined impact of hormonal imbalance, metabolic stress, and hypovitaminosis D [95]. Intervention studies on vitamin D supplementation in PCOS show promising but sometimes variable outcomes. Supplementation, often alongside calcium or metformin, has been reported to improve menstrual regularity, reduce AMH levels, lower fasting glucose, and enhance HOMA-IR [96]. Meta-analyses suggest modest improvements in lipid profiles and potential reductions in hyperandrogenism [97]. Daily supplementation may be more effective for glycemic control than weekly dosing, with the greatest benefits seen in those with baseline deficiency. Effects on BMI, triglycerides, HDL cholesterol, and dehydroepiandrosterone sulfate (DHEA-S) are less consistent, and some trials show no significant metabolic or hormonal changes despite correcting vitamin D levels [98]. Importantly, supplementation is safe, low-cost, and may serve as an effective adjunct therapy.

## 3. The Role of Vitamin D in Cardiovascular Disease

### 3.1. Vitamin D in Hypertension

Hypertension remains one of the most prevalent and modifiable risk factors for cardiovascular morbidity and mortality worldwide [99]. Emerging evidence suggests that vitamin D may influence both the development and progression of elevated blood pressure through several biological pathways, including modulation of the renin–angiotensin–aldosterone system (RAAS), regulation of endothelial function, control of vascular smooth muscle cell (VSMC) activity, maintenance of calcium-phosphate homeostasis, and regulation of inflammation [100,101,102]. Vitamin D regulates blood pressure via genomic and non-genomic mechanisms, both primarily mediated by VDR, as well as through indirect effects on calcium–phosphate balance [103,104].

The active form of vitamin D, 1,25(OH)_2_D (calcitriol), exerts genomic effects by binding to the intracellular VDR in a ligand-dependent manner. As outlined in the Introduction, calcitriol modulates the expression of genes involved in RAAS regulation, vascular tone, and inflammatory responses [105]. Calcitriol reduces plasma renin activity and angiotensin II (Ang II) concentrations. Consequently, lower Ang II levels may limit vasoconstriction, sodium retention, and sympathetic nervous system activation, thereby contributing to reduced peripheral vascular resistance and blood pressure [106]. This mechanism is supported by experimental data. In VDR knockout mice, renal renin mRNA expression increased more than threefold, and plasma Ang II levels rose more than 2.5-fold compared to wild-type controls (*p* < 0.001), leading to hypertension, cardiac hypertrophy, and increased water intake. Pharmacological inhibition of calcitriol synthesis in wild-type mice significantly increased renin expression (*p* < 0.01), whereas exogenous calcitriol administration reduced renin levels by about 50% after five doses (*p* < 0.05) [107]. Genomic actions of calcitriol upregulate endothelial nitric oxide synthase (NOS3), increasing nitric oxide (NO) bioavailability, which promotes vasodilation and improves endothelial function. Additionally, it reduces oxidative stress by enhancing the expression of antioxidant enzymes and activating the nuclear factor erythroid 2–related factor 2 (Nrf2) antioxidant pathway, thereby supporting NO-mediated vascular protection. Calcitriol also exerts anti-inflammatory effects by downregulating pro-inflammatory cytokines, particularly IL-1, and broadly suppressing other inflammatory mediators such as TNF-α and MCP-1. These effects involve inhibition of intracellular pathways such as NF-κB and STAT, ultimately contributing to improved endothelial function and arterial compliance [92,108,109].

Calcitriol also exerts rapid, non-genomic effects via membrane-associated receptors such as the 1,25-dihydroxyvitamin D_3_–membrane-associated rapid response steroid-binding protein (1,25D-MARRS) [110]. Activation of these receptors triggers intracellular signaling cascades involving MAPK, particularly extracellular signal-regulated kinases 1 and 2 (ERK1/2), PI3K, and protein kinase C (PKC) pathways [111]. These cascades increase intracellular Ca^2+^ and directly phosphorylate NOS3, enhancing NO synthesis and promoting rapid vasodilation [100]. Although these effects occur within seconds to minutes, they may also influence gene expression via transcription factors such as specificity proteins 1 and 3 (SP1/3) and RXR, potentially contributing to longer-term vascular remodeling [6,101].

Calcitriol contributes to long-term blood pressure regulation through its central role in calcium–phosphate balance. Both genomic and non-genomic pathways are involved in this regulation. Genomically, calcitriol upregulates intestinal and renal calcium transporters such as transient receptor potential vanilloid channels (TRPV5 and TRPV6), calbindin-D_9_k, and plasma membrane Ca^2+^-ATPase (PMCA1b), enhancing dietary calcium absorption and renal calcium reabsorption [112,113]. Non-genomic actions via membrane VDR or 1,25D-MARRS increase calcium influx in enterocytes and renal tubular cells [6]. Calcitriol modulates phosphate homeostasis by stimulating intestinal phosphate uptake via the NaPi-2b (Slc34a2) cotransporter and regulating PTH secretion [114]. This interplay plays a vital role in maintaining systemic phosphate balance. Chronic elevation in PTH has been associated with increased arterial stiffness and vascular resistance, which are major contributors to the development of hypertension [115].

In a 12-month, double-blind RCT, Rahme M. et al. (2024) [116] studied 221 participants aged ≥65 years with overweight (BMI > 25) and vitamin D insufficiency (serum 25(OH)D: 10–30 ng/mL). Participants received daily oral vitamin D_3_ at doses of 600 IU or 3750 IU, both combined with calcium (1000 mg/day). After 12 months, the overall cohort experienced significant reductions in systolic blood pressure (SBP) (−3.5 mmHg; *p* = 0.005) and diastolic blood pressure (DBP) (−2.8 mmHg; *p* = 0.002). Although the high-dose group showed numerically greater reductions (SBP −4.2 mmHg; *p* = 0.023; DBP −3.0 mmHg; *p* = 0.01), between-group differences were not statistically significant. Effects were most pronounced in participants with BMI > 30 and/or baseline hypertension [116].

Similarly, a double-blind RCT of 208 patients aged 26–84 with essential hypertension and vitamin D deficiency (serum 25(OH)D < 20 ng/mL) or insufficiency (20–30 ng/mL) randomized participants to receive vitamin D pearls—50,000 IU weekly for two months in deficient patients or 1000 IU weekly for two months in insufficient patients—or placebo, alongside usual antihypertensive therapy. Blood pressure was measured at baseline and after one and two months. After one month, SBP significantly decreased in both the vitamin D group (from 152.93 to 134.93 mmHg) and the placebo group (from 149.45 to 138.37 mmHg), with a greater reduction in the vitamin D group (*p* = 0.003). This effect persisted at two months, with mean SBP of 127.65 mmHg in the vitamin D group versus 130.97 mmHg in placebo (*p* = 0.037). No significant differences in DBP were observed at either time point [117].

A meta-analysis by Zhang D et al. examined the relationship between serum 25(OH)D levels and hypertension risk, as well as the effect of vitamin D supplementation on blood pressure in healthy adults aged 18 and older without cardiovascular or metabolic diseases. It included 11 cohort studies (43,320 participants) and 27 RCTs (3810 subjects). The dose–response analysis revealed a nonlinear association: hypertension risk increased sharply below 75 nmol/L of 25(OH)D and stabilized but remained significant between 75 and 130 nmol/L. However, pooled RCT results showed no significant effect of vitamin D supplementation on systolic (weighted mean difference [WMD] −0.00 mm Hg; 95% CI −0.71 to 0.71) or diastolic blood pressure (WMD 0.19 mm Hg; 95% CI −0.29 to 0.67) [118].

In a 2025 systematic review and meta-analysis, Amer SA et al. synthesized evidence from eight RCTs evaluating the effects of vitamin D_3_ supplementation on blood pressure in adults with mild to moderate hypertension. Intervention regimens encompassed a broad range of dosing strategies, from daily supplementation with 1000–3000 IU to intermittent high-dose boluses of 50,000–200,000 IU, administered weekly, monthly, or as single doses, with treatment durations ranging from 1 week to 18 months. Compared with placebo, pooled analysis demonstrated a statistically significant, albeit modest, reduction in SBP (−2.83 mmHg; *p* = 0.04) and DBP (−1.64 mmHg; *p* = 0.01). The most pronounced effects were observed in individuals with greater baseline vitamin D deficiency and/or elevated baseline blood pressure [119].

Another randomized, double-blind, placebo-controlled trial involving 200 hypertensive patients with serum 25(OH)D < 30 ng/mL compared supplementation with 2800 IU vitamin D_3_ daily for 8 weeks against placebo. Ambulatory blood pressure monitoring (ABPM) showed no significant effects on 24-h SBP or DBP in any subgroup, including those with severe deficiency (<20, <16, and <12 ng/mL). An exploratory analysis indicated a marginally significant inverse association between achieved 25(OH)D levels and 24-h SBP (*p* = 0.003), but this finding should be interpreted cautiously due to small subgroup sizes and post hoc design [120].

Clinical trials on vitamin D supplementation and blood pressure have yielded inconsistent results. Some evidence suggests modest reductions in overweight, obese, or hypertensive individuals with vitamin D deficiency or insufficiency [106]. However, larger meta-analyses and trials in healthy adults without cardiovascular or metabolic diseases show no significant overall effect [108]. These results are summarized in Table 2. Variability in outcomes may reflect differences in baseline vitamin D status, supplementation dosage, treatment duration, and participant characteristics. Further well-powered, targeted studies are necessary to clarify the role of vitamin D in blood pressure regulation and to identify populations that may benefit most from supplementation [121].

### 3.2. Vitamin D in Coronary Artery Disease (CAD) and Atherosclerosis

Atherosclerosis, the primary pathological basis of coronary artery disease (CAD), is a chronic, progressive inflammatory condition characterized by lipid accumulation, immune cell activation, and remodeling of the arterial wall. These processes collectively lead to plaque formation, luminal narrowing, and clinical manifestations such as myocardial infarction (MI) and angina [122]. Vitamin D has emerged as a potential modulator of multiple biological pathways involved in atherosclerosis, including endothelial function, vascular inflammation, lipid metabolism, and vascular calcification, indicating a possible role in both the prevention and progression of CAD [123].

One of the key mechanisms by which vitamin D may exert its protective effects is through suppression of vascular inflammation, a fundamental driver of atherosclerotic disease. Vitamin D downregulates pro-inflammatory cytokines such as IL-6 and TNF-α, thereby reducing endothelial activation, leukocyte adhesion, and foam cell formation, all critical processes in plaque development [124]. Moreover, vitamin D inhibits pivotal signaling pathways including NF-κB and MAPK, resulting in decreased expression of pro-inflammatory genes and enhanced plaque stabilization [29]. Additionally, vitamin D promotes the expansion of regulatory T cells, which counterbalance pro-inflammatory Th1 and Th17 subsets that contribute to vascular injury. This immunomodulatory effect is particularly relevant given the predominance of Th1 cells in atherosclerotic plaques, which secrete interferon-gamma (IFN-γ) and express the Th1 master transcription factor T-bet, driving inflammation, lesion progression, and plaque instability [125,126].

Vitamin D also influences lipid metabolism, a critical factor in atherogenesis. It facilitates cholesterol efflux from macrophages by upregulating ATP-binding cassette transporter A1 (ABCA1), thereby limiting foam cell formation [127]. Furthermore, vitamin D modulates enzymes involved in lipid processing, such as hepatic lipase and lipoprotein lipase, contributing to a more favorable lipid profile and reduction in low-density lipoprotein cholesterol (LDL-C) [113]. Observational data support these associations; for instance, a large cross-sectional study by Gholamzad et al., including 15,600 healthy adults, demonstrated a significant inverse relationship between serum 25(OH)D levels and LDL-C (*p* < 0.05) [128]. Similarly, Li et al. reported that an annual increase in serum 25(OH)D of ≥10 ng/mL correlated with a statistically significant LDL-C reduction of approximately 7–9 mg/dL, independent of traditional cardiovascular risk factors [129]. RCTs investigating the lipid-modulatory effects of vitamin D supplementation have yielded mixed results. Serrano et al. found a significant LDL-C reduction of –11.5 mg/dL (*p* = 0.030) in healthy young adults receiving 1000 IU vitamin D daily for 15 weeks, compared to 200 IU [130]. Conversely, a recent meta-analysis by Lu et al., encompassing 20 RCTs with 1711 participants with type 2 diabetes, identified significant increases in high-density lipoprotein cholesterol (HDL-C; +1.6 mg/dL, *p* = 0.03) and decreases in triglycerides (−8.6 mg/dL, *p* = 0.01) but found no significant effects on LDL-C or total cholesterol. The meta-analysis also suggested that individuals with lower baseline BMI and higher baseline 25(OH)D levels experienced greater lipid profile improvements, and that higher doses and shorter supplementation durations were associated with more favorable outcomes [131].

**Figure 3:**
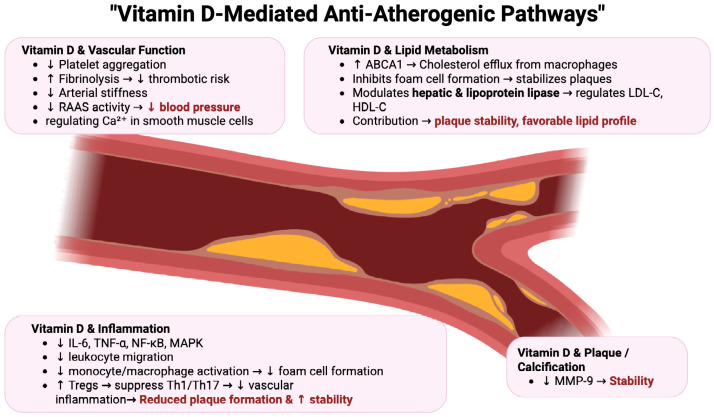
Vitamin-D-Mediated Anti-Atherogenic Pathways in Cardiovascular Protection [29,123,124,125,126,127]. Vitamin D exerts multifaceted protective effects against atherosclerosis by modulating inflammation, endothelial function, lipid metabolism, plaque stability, and thrombosis. These coordinated actions reduce vascular inflammation, improve endothelial health, promote favorable lipid profiles, stabilize plaques, and lower thrombotic risk, ultimately decreasing the progression of atherosclerosis and reducing cardiovascular events. Abbreviations: ABCA1—ATP-binding cassette transporter A1, Ca^2+^—calcium ion, HDL-C—high-density lipoprotein cholesterol, IL-6—interleukin 6, LDL-C—low-density lipoprotein cholesterol, MAPK—mitogen-activated protein kinase, MMP-9—matrix metalloproteinase 9, NF-κB—nuclear factor kappa-light-chain-enhancer of activated B cells, RAAS—renin-angiotensin-aldosterone system, Tregs—regulatory T cells, TNF-α—tumor necrosis factor alpha, Th1/Th17—T helper 1 and T helper 17 cells.

Vitamin D may also contribute to plaque stability. Foroughinia et al. conducted a cross-sectional study involving 150 patients undergoing elective percutaneous coronary intervention (PCI), demonstrating a significant inverse correlation between serum 25(OH)D and matrix metalloproteinase-9 (MMP-9) levels (*p* = 0.039), a biomarker linked to plaque vulnerability and adverse cardiovascular remodeling [132]. Regarding vascular calcification (VC), observational evidence indicates that low serum 25(OH)D concentrations are associated with increased coronary artery calcium scores (CACS) and greater luminal stenosis severity on coronary computed tomography angiography (CCTA). Pella et al. reported a significant inverse correlation between serum 25(OH)D and CACS (*p* < 0.0001), with vitamin D deficiency linked to markedly higher coronary calcification compared to sufficient vitamin D status [133]. However, a recent Mendelian randomization study by Meena et al., leveraging genome-wide association data from over 400,000 individuals of European descent, found no causal association between genetically predicted 25(OH)D levels and carotid artery plaque, carotid intima-media thickness (cIMT), or coronary artery calcium score (CACS) [134]. The VITAL trial, involving over 25,000 participants, assessed the effects of daily vitamin D_3_ supplementation (2000 IU/day) and marine n-3 fatty acids (1 g/day) on cardiovascular outcomes over a median follow-up of 5.3 years, and found no significant reduction in major cardiovascular events compared with placebo [135]. Similarly, the ViDA trial, including over 5000 adults aged 50–84 years, reported that monthly high-dose vitamin D_3_ supplementation (100,000 IU) did not lower the incidence of cardiovascular disease events during a median follow-up of 3.3 years [136]. These findings are consistent with a recent meta-analysis of 18 RCTs with long-term vitamin D supplementation (≥1 year), which found no significant reduction in major adverse cardiovascular events (MACE), including MI, heart failure, cardiovascular death, or all-cause mortality. Collectively, these data indicate that vitamin D supplementation alone is unlikely to provide consistent cardiovascular protection in the general population. Nevertheless, targeted supplementation in well-defined high-risk groups—such as individuals with severe vitamin D deficiency—may hold clinical relevance. Future investigations should prioritize these subgroups to determine whether personalized supplementation strategies can yield meaningful cardiovascular benefit [137]. As presented in Figure 3, the vitamin-D-mediated anti-atherogenic pathways contribute to cardiovascular protection, including modulation of inflammation, endothelial function, lipid metabolism, plaque stability, and thrombotic risk.

## 4. Vitamin D in Chronic Kidney Disease

Kidney disease is a growing global health issue linked to high individual, healthcare and societal costs. It is estimated that around 700 million people worldwide are living with CKD [138]. According to a meta-analysis of observational studies by Hill N. et al., approximately 13.4% of the global population is affected by CKD [139]. In high-income countries, CKD occurs in roughly one in three individuals with diabetes and one in five individuals with hypertension [138]. CKD increases the risk of adverse outcomes, most notably progression to end-stage kidney disease (ESKD) requiring renal replacement therapy such as dialysis or renal transplantation [140].

CKD has multiple causes, including diabetes mellitus, glomerulonephritis, genetic disorders, cardiovascular and multisystem diseases, medications, urological disorders, infections, and acute kidney injury. Diabetes mellitus is the leading cause of CKD worldwide, with about 40% of patients developing the disease. Annual assessment of GFR and urine albumin–creatinine ratio is recommended for all individuals with diabetes. Optimal glycaemic control, early antihypertensive therapy with an angiotensin-converting enzyme inhibitor or angiotensin receptor blocker and treatment with a sodium glucose co-transporter inhibitor are important for slowing CKD progression [140].

Patients with CKD have an increased risk of metabolic and cardiovascular diseases. As reported by Jankowski J. et al., individuals with CKD face a heightened cardiovascular risk, manifesting as coronary artery disease, heart failure, arrhythmias and sudden cardiac death [141]. Although the incidence and prevalence of cardiovascular events are already significantly higher in early stages of CKD compared to the general population, those with advanced disease face a substantially greater risk. In this high-risk group, cardiovascular disease, rather than ESKD, is the leading cause of death. Hypertension is a common consequence of CKD, contributes to its progression and may independently increase the risk of ESKD. All patients with newly diagnosed hypertension should be screened for CKD and blood pressure control is important to slow disease progression [140].

A decline in GFR, a hallmark of disease progression, is associated with the development of serious complications such as arterial hypertension, anemia, hyperkalemia, metabolic acidosis and mineral bone disorders. Among these, mineral bone disorders are particularly important, as their presence and inadequate management are linked to a greatly increased risk of death, cardiovascular events and further progression of CKD to ESKD [142].

Vitamin D is increasingly recognized as a renoprotective factor in CKD, acting through multiple overlapping molecular and systemic pathways [143]. Under normal physiology, vitamin D undergoes hydroxylation in the liver to form 25(OH)D, which is further converted in the kidney by 1α-hydroxylase into its active form, 1,25(OH)_2_D [144]. In CKD progressive nephron loss, reduced renal mass and diminished delivery of substrate to 1α-hydroxylase impair this conversion, resulting in falling levels of circulating active vitamin D even at early stages of disease [145]. Compounding this, tubular uptake of vitamin-D-binding protein via the receptor megalin is reduced, leading to urinary losses of 25(OH)D and worsening systemic deficiency. The disruption of vitamin D metabolism is further aggravated by elevated fibroblast growth factor 23 (FGF-23), a phosphaturic hormone that rises as GFR declines [146]. FGF-23 suppresses renal 1α-hydroxylase activity, induces 24-hydroxylase and accelerates degradation of both 25(OH)D and 1,25(OH)_2_D. Persistent phosphate retention, increased PTH fragments and uremic toxins also contribute to the suppression of vitamin D activation. These changes form a vicious cycle in which vitamin D deficiency not only reflects CKD progression but also actively accelerates it [147].

Vitamin D and its analogues exert renoprotective effects by targeting several pathological pathways that drive CKD. Vitamin D suppresses the RAAS, thereby lowering intraglomerular pressure, reducing proteinuria and alleviating glomerular injury. In animal models, administration of calcitriol or paricalcitol decreases renin and angiotensin II expression, attenuating proinflammatory and profibrotic signaling downstream of RAAS activation. Vitamin D acts as a potent anti-inflammatory agent by inhibiting NF-κB, a transcription factor responsible for upregulating cytokines, chemokines, and adhesion molecules such as TNF-α, IL-6, and MCP-1. By inducing VDR to interact with NF-κB p65 subunits, vitamin D prevents transcription of inflammatory mediators, thereby reducing leukocyte infiltration and tubular injury. Moreover, vitamin D has strong antifibrotic actions [148]. It interferes with transforming growth factor-β (TGF-β)/Smad and Wnt/β-catenin signaling, two central pathways in epithelial-to-mesenchymal transition (EMT) and interstitial fibrosis. Experimental models show that paricalcitol prevents TGF-β1–induced EMT, suppresses extracellular matrix deposition, and limits glomerulosclerosis. It also sequesters β-catenin in VDR complexes, blocking its nuclear transcriptional activity and attenuating fibrogenesis [149]. Vitamin D has antiapoptotic and cytoprotective effects. It reduces proapoptotic markers such as phospho-p53, p21, Bax, and cleaved caspase-3, while upregulating the PI3K/Akt survival pathway, thereby protecting podocytes and tubular cells from toxic, ischemic, or inflammatory injury. In experimental nephropathy, vitamin D analogues consistently lower apoptosis and preserve renal cellular integrity [150].

Vitamin D deficiency in CKD is not merely a biochemical abnormality but plays an important role in the disease course. It is highly prevalent across all CKD stages and is associated with multiple adverse outcomes, including increased mortality risk [149]. Lower vitamin D status correlates with increased proteinuria, left ventricular hypertrophy, vascular calcification, immune dysfunction, and higher susceptibility to infections. Importantly, deficiency is independently associated with all-cause and cardiovascular mortality [151]. A meta-analysis by Pilz et al. reported a 14% decrease in relative risk of death for every 10 ng/mL increase in serum 25(OH)D, underscoring the prognostic importance of vitamin D status in CKD. Intervention studies provide additional insights: supplementation with active vitamin D analogues such as paricalcitol has been shown to reduce albuminuria by up to 16% compared with placebo, especially when combined with RAAS blockade. In dialysis patients, vitamin D therapy is associated with reduced all-cause and cardiovascular mortality, though definitive randomized evidence in pre-dialysis CKD remains limited. Beyond renal outcomes, vitamin D also mitigates cardiovascular complications by attenuating myocardial hypertrophy, modulating immune responses and lowering systemic inflammation [152].

Vitamin D deficiency in CKD can be a consequence of impaired metabolism, but it also serves as a driver of renal and cardiovascular pathology. Its deficiency exacerbates RAAS activation, inflammation, fibrosis, and apoptosis, all of which hasten the decline in kidney function and increase mortality risk [149]. Conversely, supplementation with active vitamin D or its analogues can counteract these processes, providing renoprotective, antifibrotic, anti-inflammatory and cardioprotective benefits. While strong observational and experimental data support these protective roles, further well-designed randomized controlled trials are essential to establish optimal dosing, duration, and patient selection to maximize the therapeutic potential of vitamin D in CKD management [149]. Patients with CKD often have a more pronounced deficiency and insufficiency of vitamin D compared to healthy individuals and frequently require supplementation, mainly with cholecalciferol and calcifediol [153]. The progressive decline in the GFR is associated with vitamin D deficiency [154].

Vitamin D deficiency and insufficiency result from a wide range of factors. In CKD patients, causes and risk factors include older age, female sex, adiposity, proteinuria, low physical activity, peritoneal dialysis, diabetes mellitus, reduced vitamin D receptor expression, impaired tubular reabsorption, decreased skin synthesis, use of calcineurin inhibitors and reduced hepatic CYP450 activity associated with secondary hyperparathyroidism (SHPT) [155]. Beyond CKD, additional contributors include premature birth, darker skin pigmentation, limited sun exposure, obesity, malabsorption, bacterial infections, autoimmune disorders and cancer. Recent research has also identified vitamin D deficiency as a risk factor for increased mortality both in the general population and in patients with ESKD [156].

A study by Nigwekar S. et al. reported that vitamin D levels below 30 ng/mL were present in 71% of patients with CKD stage G3a/b, 84% of those in stage G4 and 89% of patients in stage G5 [157]. A study conducted by Çankaya E. et al. involving 169 patients demonstrated that vitamin D levels are lower in individuals undergoing hemodialysis and peritoneal dialysis compared to those who have undergone renal transplantation, due to various factors. The mean 25(OH)D levels were 12.74 ± 10.24 ng/mL in renal transplant patients and 11.16 ± 12.25 ng/mL in predialysis patients. In contrast, the levels were lower in patients on hemodialysis and peritoneal dialysis, at 7.77 ± 6.71 ng/mL and 5.96 ± 4.87 ng/mL, respectively [156].

Prolonged insufficiency of 25(OH)D and 1,25(OH)_2_D contributes to bone damage in CKD, commonly observed as chronic kidney disease–mineral and bone disorder (CKD-MBD), often mediated by SHPT [154].

Low 25(OH)D levels have been linked to increased bone turnover, reduced bone mineral density, and higher prevalence of SHPT in patients with CKD and those undergoing dialysis [155]. Specific studies have shown associations with central obesity and metabolic syndrome in this population [158], as well as more severe vascular calcifications in hemodialysis patients with 25(OH)D levels below 20 ng/mL [159]. According to the study by Lai S. et al., among 67 CKD patients with CKD (GFR ≥ 30 mL/min), IR and vitamin D deficiency were independently associated with left ventricular hypertrophy and atherosclerosis, highlighting their role as contributors to increased cardiovascular risk in this population [160].

The Kidney Disease: Improving Global Outcomes KDIGO 2024 Clinical Practice Guideline for the Evaluation and Management of Chronic Kidney Disease notes that, despite evidence suggesting no benefit on clinical outcomes, vitamin D supplementation and the use of calcimimetics remain common strategies to control PTH levels and maintain normal calcium levels [161]. The 2017 Kidney Disease: Improving Global Outcomes (KDIGO) guidelines on CKD-MBD suggest measuring 25(OH)D levels in patients with CKD G3a–G5D, with repeat testing based on baseline values and therapeutic interventions and recommend correcting vitamin D deficiency and insufficiency using treatment strategies advised for the general population [162].

Vitamin D supplementation in CKD remains debated areas in nephrology, with guidelines, clinical trials, and expert opinions often pointing in different directions [163].

The 2009 KDIGO guidelines suggested that in patients with CKD G3a–G5 and persistently elevated PTH, treatment with calcitriol or vitamin D analogues could be considered, whereas the 2017 KDIGO update took a more restrictive stance, recommending that active vitamin D analogues not be used routinely, but rather reserved for CKD G4–G5 patients with severe and progressive SHPT. This change was largely influenced by the PRIMO and OPERA studies, which failed to demonstrate cardiovascular benefits of paricalcitol in moderate CKD and instead raised concerns about increased hypercalcemia and potential vascular calcification. On the other hand, many nephrologists argue that this restrictive approach may delay intervention, allowing SHPT and parathyroid hyperplasia to progress, which then becomes more difficult to control [161].

Conflicting evidence also extends to the definition of vitamin D sufficiency and treatment targets. The Institute of Medicine sets sufficiency at ≥20 ng/mL (50 nmol/L), whereas the Endocrine Society, National Kidney Foundation, and several European societies recommend levels ≥30 ng/mL to optimize skeletal and possibly extra-skeletal effects [159]. Observational studies in CKD populations suggest that 25(OH)D < 20 ng/mL is associated with higher mortality, faster CKD progression, and more fractures, yet randomized controlled trials have not consistently shown improved hard outcomes with supplementation [161]. However, most randomized controlled trials on vitamin D therapy in the CKD population have primarily evaluated surrogate biochemical markers rather than clinical outcomes, with clinical endpoints typically reported as adverse events rather than predefined trial outcomes [162].

According to a systematic review and meta-analysis by Christodoulou M. et al., vitamin D supplementation in CKD patients increases 25(OH)D levels, but the dose–response appears attenuated compared with healthy individuals, and the effects on PTH remain inconsistent. While calcitriol and paricalcitol consistently suppress PTH, calcifediol may represent a promising alternative with a lower risk of hypercalcemia, although comparative meta-analyses are still needed. The potential increase in FGF23 with vitamin D analogues underscores the need for further research to clarify their differential effects on mineral metabolism and cardiovascular risk in CKD [159].

Dosing strategies remain controversial, while The Institute of Medicine defines the upper safe intake at 4000 IU/day, some experts advocate for higher doses in overweight and obese patients due to sequestration of vitamin D in adipose tissue, but concerns about hypercalcemia, hyperphosphatemia, kidney stones and FGF-23 elevation remain unresolved [163].

Another point of conflict is whether native vitamin D (cholecalciferol, ergocalciferol, calcifediol) is sufficient for CKD patients, or whether active forms (calcitriol, paricalcitol, doxercalciferol) are needed earlier in disease progression. Trials with native vitamin D have shown modest improvements in PTH, bone mineral density, and inflammation, but inconsistent results on CKD progression and mortality. Active forms are more potent but carry higher risks of hypercalcemia and oversuppression of PTH, which may contribute to adynamic bone disease and adverse cardiovascular effects. The lack of standardized outcome measures (fracture, GFR decline, cardiovascular events) and heterogeneous trial designs (different doses, formulations, follow-up times) further complicate interpretation [164].

In summary, controversy persists over optimal thresholds, timing, and formulations of vitamin D supplementation in CKD. While most agree that vitamin D deficiency should be corrected, the safety and benefit of aiming for higher levels, the choice between native and active analogues, and the impact on long-term outcomes remain unresolved. The 2017 KDIGO update reflects caution after negative trials, but many experts caution that this approach may be overly conservative [162]. Ongoing debates highlight the urgent need for large, well-designed RCTs that evaluate not only biochemical targets but also hard clinical endpoints such as fractures, cardiovascular events, and CKD progression [165]. Until then, practice will likely remain heterogeneous, balancing the risk of deficiency against the risks of overtreatment [162].

## 5. Discussion

Numerous studies have linked serum 25(OH)D levels to a broad range of disorders, including musculoskeletal, metabolic, cardiovascular, malignant, autoimmune and infectious diseases [166,167]. Vitamin D receptors are widely expressed in organs such as the pancreas, intestines, muscles, and nervous system. By binding to these receptors, vitamin D regulates the cell cycle and modulates the activity of immune, nervous and cardiovascular cells. In the kidneys, it exerts protective effects by attenuating renal fibrosis, reducing inflammation, and slowing the progression of proteinuria [148].

Accordingly, maintaining adequate vitamin D levels has been proposed as a safe and cost-effective strategy for lowering the risk of chronic non-skeletal diseases and overall mortality [168].

Ongoing research continues to explore the effects of vitamin D deficiency and supplementation across diverse conditions. While causal relationships remain unproven, these associations have driven widespread supplementation and increased 25(OH)D testing [169].

Vitamin D supplementation may involve several compounds, including cholecalciferol, ergocalciferol, calcidiol and calcitriol. Of these, only cholecalciferol represents the natural and biologically relevant form for nutritional purposes. Ergocalciferol is synthetic, less stable, and less potent, while calcidiol and calcitriol act primarily as biomarkers or hormones rather than nutrients [170]. Recent evidence also indicates that ergocalciferol is prone to degradation during storage and cooking, supporting the use of cholecalciferol as the preferred form for dietary supplementation and food fortification [171].

The 2024 Endocrine Society Clinical Practice Guideline on Vitamin D for Disease Prevention recommends empiric supplementation for children and adolescents (1–18 years) to prevent rickets and reduce respiratory infections; adults ≥ 75 years to lower overall mortality. Pregnant women to reduce pregnancy-related complications; and individuals with high-risk prediabetes to slow progression to diabetes. Daily supplementation is preferred for adults over 50 years, whereas doses above the Dietary Reference Intake are not recommended for healthy adults under 75 years. Overall, empiric supplementation is considered safe, feasible and may contribute to improved cardiovascular and metabolic health [169].

From a public health perspective, vitamin D deficiency remains highly prevalent worldwide, affecting up to 7% of the global population with severe deficiency without access to supplementation [172]. High-risk groups include older adults, individuals with obesity, those with limited sun exposure or darker skin, and patients with chronic illnesses such as malabsorption syndromes, chronic kidney disease, or liver disease [169,173,174]. Food fortification of staple foods, combined with targeted supplementation in risk groups, is a cost-effective and scalable strategy to improve vitamin D status and lessen the overall population burden of deficiency [175]. By contrast, routine population-wide 25(OH)D testing is not cost-effective and is not recommended; assessment should be reserved for clearly defined risk groups or clinical indications [169]. Overall, a pragmatic approach integrating fortification, targeted supplementation, and selective testing maximizes health benefits while conserving healthcare resources [169,173,174].

Epidemiological studies highlight the magnitude of deficiency. In Europe, a study by Cashman et al. (2016) [176] of 55,844 individuals found that 13.0% had serum 25(OH)D concentrations below 30 nmol/L on average throughout the year, with rates of 17.7% in the extended winter (October–March) and 8.3% in the summer (April–November). Using a higher threshold of <50 nmol/L, 40.4% were deficient [176]. In the United States, 24% and 6% of adults had 25(OH)D levels below 50 and 25 nmol/L, respectively [177].

Evidence on mortality outcomes remains mixed. A study by Zhang et al. (2019) [178] including 74,655 participants reported that vitamin D supplementation was not associated with all-cause mortality, cardiovascular mortality, or non-cancer, non-cardiovascular mortality compared with placebo or no treatment. However, supplementation was linked to a 15% reduction in cancer-related mortality. Subgroup analyses indicated that trials using vitamin D3 showed lower all-cause mortality than those using vitamin D2. Nevertheless, further large-scale clinical trials are needed to clarify whether vitamin D3 supplementation meaningfully reduces all-cause mortality [178]. Weaver et al. demonstrated that supplementation with calcium and vitamin D reduced the overall risk of fractures by 15% and hip fractures by 30% [179]. However, studies involving older adults have shown that high-dose vitamin D supplementation in those with normal levels does not provide significant benefits in reducing falls or fractures [180,181].

The optimal dosage of vitamin D supplementation remains controversial. The US Institute of Medicine’s Food and Nutrition Board (2010) recommended a dietary reference intake (DRI) of 400 IU/day (10 μg/day) for infants and 600 IU/day (15 μg/day) for children and adults up to 70 years, with the same value applied to adults over 70 years. These recommendations were primarily based on vitamin D’s role in calcium metabolism and the prevention of rickets. Vitamin D insufficiency and deficiency were defined as circulating levels of 52–72 nmol/L and <50 nmol/L, respectively [182].

Although vitamin D toxicity is rare, it usually results from deliberate excessive supplementation, as endogenous synthesis from sunlight and dietary intake rarely reach harmful levels. The main complication is hypercalcemia caused by excessive calcium deposition [183]. Mild, asymptomatic hypercalcemia has been observed in children receiving 1400–4000 IU/day, associated with serum 25(OH)D concentrations of 197–255 nmol/L. Severe hypercalcemia has been reported in infants exposed to very high doses, up to 60,000 IU/day [184]. In contrast, studies in healthy adults have shown that supplementation with as much as 50,000 IU of vitamin D_2_ every other week (around 3300 IU/day) over six years maintained serum 25(OH)D levels of 100–150 nmol/L without signs of toxicity [185]. Similarly, Ekwaru et al. reported that Canadian adults receiving 20,000 IU/day of vitamin D_3_ reached concentrations up to 150 nmol/L without adverse effects [186]. Importantly, the no observed adverse effect level (NOAEL) has been set at 10,000 IU/day, while the tolerable upper intake level (UL) is 4000 IU/day [182].

The interpretation of vitamin D research is subject to some limitations. Most data are derived from observational studies, which are prone to reverse causality and residual confounding. In addition, publication bias may overestimate the apparent benefits, as studies with null or negative findings are less likely to be published [187,188]. Heterogeneity also exists between studies, partly due to inter-individual variability in vitamin D metabolism, which is influenced by polymorphisms in genes such as CYP2R1 and CYP27B1 [14,189]. These genetic variants contribute to the concept of differential responders to supplementation (high vs. low responders), complicating the establishment of universal dosing guidelines. Additional variability arises from differences in baseline 25(OH)D concentrations and lifestyle factors, including diet, sunlight exposure, and adiposity [175,188,190].

These sources of variability complicate the formulation of universal supplementation guidelines. At the cellular level, additional barriers include the tissue-specific distribution of vitamin D receptors, enzymatic inactivation by CYP24A1, and sequestration of vitamin D in adipose tissue, all of which may limit its biological availability and efficacy [14,190,191]. Additionally, the development of vitamin D analogues and CYP11A1-derived metabolites, including lumisterol and tachysterol, has revealed promising antioxidant and immunomodulatory properties, broadening the potential therapeutic spectrum [14,190,192]. These biological limitations of vitamin D are summarized in Table 3.

In addition to these biological limitations, conventional oral and intramuscular supplementation is further constrained by inconsistent absorption, poor solubility, and rapid degradation. To address these shortcomings, novel nanotechnology-based delivery systems have been developed. Organic nanocarriers such as liposomes, polymeric nanoparticles, and micelles enhance vitamin D stability, allow for controlled release, and improve tissue-specific targeting. These approaches not only increase therapeutic efficacy but also enable lower dosing while minimizing systemic side effects. Moreover, encapsulation strategies permit combination with other therapeutic agents, potentially broadening the role of vitamin D beyond bone and mineral metabolism, with promising applications in oncology and immunomodulation [193].

Despite the large body of research, conclusive data on the protective role of vitamin D remain lacking, and there is no solid basis for recommending supplementation beyond established indications [187,188].

## 6. Conclusions

Vitamin D exerts far-reaching effects that extend beyond skeletal health, influencing metabolic regulation, vascular function, and immune responses. Deficiency is highly prevalent worldwide and has been consistently associated with an increased risk of major lifestyle-related diseases, including T1DM, T2DM, hypertension, CAD, CKD, and PCOS. Experimental and clinical studies highlight its role in modulating oxidative stress, inflammation, endothelial function, and hormonal signaling.

Despite substantial evidence linking low vitamin D levels with adverse metabolic and cardiovascular outcomes, the results from supplementation trials remain inconsistent. Variability in study populations, baseline vitamin D status, dosage, and duration may account for these discrepancies. Current findings suggest that maintaining adequate vitamin D concentrations is important for overall cardiometabolic health, but supplementation as a therapeutic strategy requires a more personalized approach.

Future research on vitamin D should focus on elucidating the precise molecular mechanisms underlying its antioxidant activity, including the role of VDR gene expression and its effects on cellular processes, as well as its impact on mitochondrial function, biogenesis, and oxidative stress reduction. Large-scale, long-term clinical trials are needed to evaluate the efficacy of vitamin D supplementation in preventing and managing oxidative-stress-related diseases, identify reliable biomarkers, and determine optimal dosages tailored to individual factors such as age, sex, genetics, and pre-existing conditions. Research should also explore vitamin D’s potential in modulating chronic diseases characterized by high oxidative stress—such as cardiovascular disease, type 2 diabetes, and chronic kidney disease—as well as its neuroprotective effects, immune regulation, and role in inflammatory and autoimmune conditions.

## Figures and Tables

**Table 1:** Vitamin D Receptor (VDR) gene polymorphisms and their association with Gestational Diabetes Mellitus (GDM).

Polymorphism (SNP)	Mechanism/Role	Clinical Relevance
rs7975232 (ApaI)	May alter VDR transcription and mRNA stability [71,72].	Associated with increased GDM risk in several populations; meta-analyses support link
rs2228570 (FokI)	Alters VDR protein length and activity; impacts receptor signaling [72,73,74].	Linked to higher susceptibility to GDM; associated with insulin resistance and β-cell dysfunction
rs1544410 (BsmI)	May affect mRNA stability and receptor expression [71,72,74].	Related to insulin secretion defects; linked to GDM in Saudi population
rs731236 (TaqI)	Silent polymorphism; may influence VDR expression [71,72,74].	Associated with occurrence of GDM in Saudi population (with ApaI, BsmI, FokI)
rs10783219	SNP variant shown to affect VDR function [71,72].	Significantly increased risk of GDM in genetic studies
rs739837	May influence gene regulation, but no clear mechanistic link found [71,72].	Not associated with GDM development

Abbreviations: GDM—Gestational diabetes mellitus, SNP—Single nucleotide polymorphism, VDR—Vitamin D receptor, mRNA—messenger ribonucleic acid.

**Table 2:** Summary of randomized controlled trials and meta-analyses evaluating the effects of vitamin D supplementation on blood pressure in adults.

Study/Year	Population & Baseline 25(OH)D	Intervention & Duration	Main Findings
Rahme et al. [116]	≥65 y, overweight, 25(OH)D 10–30 ng/mL	600 IU or 3750 IU/day + Ca 1000 mg/day, 12 mo	↓ SBP −3.5 mmHg, ↓ DBP −2.8 mmHg; greater benefit in obese/hypertensive
Sheikh et al. [117]	26–84 y, essential HTN, 25(OH)D < 20 ng/mL (deficiency) or 20–30 ng/mL (insufficiency)	50,000 IU/week × 8 wk (deficient) or 1000 IU/week × 8 wk (insufficient)	Greater ↓ SBP vs. placebo at 1 and 2 mo; DBP NS
Grübler et al. [120]	HTN, 25(OH)D < 30 ng/mL	2800 IU/day × 8 wk	No overall BP effect; inverse association between achieved 25(OH)D and SBP
Amer et al. (meta-analysis) [119]	Mild–moderate HTN	8 RCTs, doses 1000–3000 IU/day or 50,000–200,000 IU bolus, duration 1 wk–18 mo	↓ SBP −2.83 mmHg, ↓ DBP −1.64 mmHg; greatest effect in deficiency/high BP
Zhang et al. (meta-analysis) [118]	Healthy adults ≥ 18 y	27 RCTs	No BP effect; cohort data show higher HTN risk below 75 nmol/L 25(OH)D

Abbreviations: SBP—systolic blood pressure; DBP—diastolic blood pressure; NS—not significant; HTN—hypertension; 25(OH)D—25-hydroxyvitamin D; mo—months; wk—weeks; y—years; RCT—randomized controlled trial.

**Table 3:** Biological limitations of vitamin D activity [14,175,189,190,191,192].

Limitation	Mechanism	Clinical/Research Implications
Tissue-specific VDR expression	Vitamin D activity depends on VDR distribution, which varies between tissues	Uneven effects across organs; limited responses in tissues with low VDR density
CYP24A1-mediated degradation	Rapid catabolism of active vitamin D metabolites by CYP24A1 reduces intracellular availability	May blunt therapeutic efficacy; rationale for developing analogues resistant to CYP24A1
Sequestration in adipose tissue	Vitamin D is fat-soluble and can be stored in adipose tissue, lowering circulating 25(OH)D bioavailability	Reduced effectiveness in obese individuals; higher doses may be needed
Genetic variability	Polymorphisms in CYP2R1, CYP27B1, and VDR contribute to variable metabolism	High vs. low responders; complicates universal dosing and supports personalized supplementation
Sunlight and dietary dependence	Endogenous synthesis and food-derived vitamin D depend on lifestyle and geography	Seasonal/regional differences in deficiency prevalence; supplementation or fortification often required

## Data Availability

No new data were created or analyzed in this study. Data sharing is not applicable to this article.

## Abbreviations

The following abbreviations are used in this manuscript:

1,25(OH)_2_D	1,25-dihydroxyvitamin D
1,25(OH)_2_D_3_	1,25-dihydroxyvitamin D_3_
25(OH)D	25-hydroxyvitamin D
25(OH)D_3_	25-hydroxyvitamin D_3_
7-DHC	7-dehydrocholesterol
ABCA1	ATP-binding cassette transporter 1
ABPM	Ambulatory blood pressure monitoring
AGEs	Advanced glycation end products
AMH	Anti-Müllerian hormone
Ang II	Angiotensin II
APS	Antiphospholipid syndrome
AST	Aspartate aminotransferase (if needed)
BMI	Body mass index
CACS	Coronary artery calcium score
CAD	Coronary artery disease
CAMP	Cyclic adenosine monophosphate
CCTA	Coronary computed tomography angiography
CD40/CD80/CD86/CD68	Co-stimulatory molecules on antigen-presenting cells
cIMT	Carotid intima-media thickness
CKD	Chronic kidney disease
CKD-MBD	Chronic kidney disease-mineral and bone disorder
CRP	C-reactive protein
CVD	Cardiovascular diseases
CXCL10	C-X-C motif chemokine ligand 10 (IP-10)
DAG	Diacyloglycerol
DBP	Diastolic blood pressure/Vitamin-D-binding protein (context-dependent)
DHEA-S	Dehydroepiandrosterone sulfate
DPN	Diabetic peripheral neuropathy
DRI	Dietary reference intake
EMT	Epithelial to mesenchymal transition
ESKD	End-stage kidney disease
FGF23	Fibroblast growth factor 23
FSH	Follicle-stimulating hormone
GDM	Gestational diabetes mellitus
GFR	Glomerular filtration rate
GLUT4	Glucose Transporter Type 4
HDL	High-density lipoprotein
HDL-C	High-density lipoprotein cholesterol
HbA1c	Glycated hemoglobin
HOMA-B	Homeostatic model assessment of β-cell function
HOMA-IR	Homeostatic model assessment of insulin resistance
HT	Hashimoto’s thyroiditis
HTN	Hypertension
IFN-γ	Interferon gamma
IL-1/IL-6/IL-10	Interleukin-1/Interleukin-6/Interleukin-10
IRS	Insulin receptor substrate
KDIGO	Kidney Disease: Improving Global Outcomes
LEAD	Lower extremity arterial disease
LDL	Low-density lipoprotein
LDL-C	Low-density lipoprotein cholesterol
MACE	Major adverse cardiovascular events
MAPK	Mitogen-activated protein kinases
MCP-1	Monocyte chemoattractant protein 1
MHC	Major histocompatibility complex
MI	Myocardial infarction
MMP-9	Matrix metalloproteinase 9
mo	Months
MetS	Metabolic syndrome
MS	Multiple sclerosis
mTOR	Mechanistic target of rapamycin
NF-κB	Nuclear factor kappa-light-chain-enhancer of activated B cells/Nuclear factor kappa B
Nrf2	Nuclear factor erythroid 2-related factor
NS	Not significant
NO	Nitric oxide
NOAEL	No observed adverse effect level
NOS3	Nitric oxide synthase
PPARγ	Peroxisome proliferator-activated receptor gamma
PCI	Percutaneous coronary intervention
PCOS	Polycystic ovary syndrome
PCV	Packed cell volume (if needed)
PDIA3	Protein disulfide-isomerase A3
PI3K	Phospatidylimositol 3-kinase
PKA	Protein kinase A
PKC	Protein kinase C
PTH	Parathyroid hormone
RA	Rheumatoid arthritis
RAAS	Renin–angiotensin–aldosterone system
RCT	Randomized controlled trial
ROC	Receiver operating characteristic curve
ROS	Reactive oxygen species
RXR	Retinoid X receptor
SBP	Systolic blood pressure
SHBG	Sex hormone-binding globulin
SHPT	Secondary hyperparathyroidism
SLE	Systemic lupus erythematosis
SNP	Single nucleotide polymorphism
T1DM	Type 1 diabetes mellitus
T2DM	Type 2 diabetes mellitus
T-bet	T-box transcription factor
TGF-β	Transforming growth factor beta
Th1/Th17	T helper cell subsets (pro-inflammatory)
TIR	Time in range (glycemic metric)
TRPV	Transient receptor potential vanilloid
UL	Tolerable upper intake level
VC	Vascular calcification
VDR	Vitamin D receptor
VDRE	Vitamin D response element
VITAL	Vitamin D and Omega-3 trial
ViDA	Vitamin D assessment study
VSMC	Vascular smooth muscle cell
UVB	Ultraviolet B radiation
wk	Week
